# Oral Rabies Vaccination in North America: Opportunities, Complexities, and Challenges

**DOI:** 10.1371/journal.pntd.0000549

**Published:** 2009-12-22

**Authors:** Dennis Slate, Timothy P. Algeo, Kathleen M. Nelson, Richard B. Chipman, Dennis Donovan, Jesse D. Blanton, Michael Niezgoda, Charles E. Rupprecht

**Affiliations:** 1 USDA/APHIS/Wildlife Services, National Rabies Management Program, Concord, New Hampshire, United States of America; 2 USDA/APHIS/Wildlife Services, National Rabies Management Program, Castleton, New York, United States of America; 3 Ontario Ministry of Natural Resources, Wildlife Research and Development Section, Rabies Research and Development Unit, Peterborough, Ontario, Canada; 4 Centers for Disease Control and Prevention, Division of Viral and Rickettsial Diseases, Rabies Section, Atlanta, Georgia, United States of America; George Washington University, United States of America

## Abstract

Steps to facilitate inter-jurisdictional collaboration nationally and continentally have been critical for implementing and conducting coordinated wildlife rabies management programs that rely heavily on oral rabies vaccination (ORV). Formation of a national rabies management team has been pivotal for coordinated ORV programs in the United States of America. The signing of the North American Rabies Management Plan extended a collaborative framework for coordination of surveillance, control, and research in border areas among Canada, Mexico, and the US. Advances in enhanced surveillance have facilitated sampling of greater scope and intensity near ORV zones for improved rabies management decision-making in real time. The value of enhanced surveillance as a complement to public health surveillance was best illustrated in Ohio during 2007, where 19 rabies cases were detected that were critical for the formulation of focused contingency actions for controlling rabies in this strategically key area. Diverse complexities and challenges are commonplace when applying ORV to control rabies in wild meso-carnivores. Nevertheless, intervention has resulted in notable successes, including the elimination of an arctic fox (*Vulpes lagopus*) rabies virus variant in most of southern Ontario, Canada, with ancillary benefits of elimination extending into Quebec and the northeastern US. Progress continues with ORV toward preventing the spread and working toward elimination of a unique variant of gray fox (*Urocyon cinereoargenteus*) rabies in west central Texas. Elimination of rabies in coyotes (*Canis latrans*) through ORV contributed to the US being declared free of canine rabies in 2007. Raccoon (*Procyon lotor*) rabies control continues to present the greatest challenges among meso-carnivore rabies reservoirs, yet to date intervention has prevented this variant from gaining a broad geographic foothold beyond ORV zones designed to prevent its spread from the eastern US. Progress continues toward the development and testing of new bait-vaccine combinations that increase the chance for improved delivery and performance in the diverse meso-carnivore rabies reservoir complex in the US.

## Introduction

Oral rabies vaccination (ORV) represents a socially acceptable methodology that may be applied on a broad geographic scale to manage the disease in specific terrestrial wildlife reservoirs, as well as in free-ranging or feral dog (*Canis familiaris*) populations, where parenteral vaccination is impractical. The integration of ORV into conventional prevention and control strategies signifies a paradigm shift toward the achievement of effective rabies control in terrestrial wild Carnivora and feral dog reservoirs. Inherent to interventions targeting specific reservoirs are the benefits associated with reduced risk of human and animal exposure to rabies. Moreover, success in geographically confining or reducing terrestrial rabies virus diversity should facilitate an enhanced focus on the public and animal health dilemma associated with rabies in bats in the US and elsewhere in North America.

To date, ORV has been successfully applied to eliminate rabies among red foxes (*Vulpes vulpes*) in several European countries [1],[2], with continued expansion of control programs into eastern Europe [3]. Ontario, Canada has nearly achieved elimination of the arctic fox (*V. lagopus*) variant of rabies virus, once widespread in red foxes throughout the southern part of the province [4],[5]. However, the virus currently persists in isolated foci in southwestern Ontario as a result of spill-over into skunks (*Mephitis mephitis*) [6] and the lack of an effective oral vaccine-bait formulation for use in skunks [7]. Development and field testing of a human adenovirus-rabies glycoprotein recombinant (ONRAB [Artemis Technologies Inc., Guelph, Ontario, Canada]) with proven effectiveness in skunks holds promise for total elimination of the arctic fox rabies variant in southern Ontario [8],[9]. ORV has also been used to successfully control a canine rabies virus variant that had spilled over into coyotes (*C. latrans*) in south Texas [10],[11]. Its integration into the control strategy as an adjunct to parenteral vaccination in dogs has been credited as a major contributing factor leading to canine rabies free status for the US (declared in 2007 based on World Health Organization [WHO] standards [12],[13]).

Currently, ORV use in the US remains focused on preventing raccoon (*Procyon lotor*) rabies from expanding its geographic foothold beyond the eastern US and Canada, and as a component of potential strategies for raccoon rabies elimination [14],[15]. Additional programs are focused on containing and eliminating a variant of gray fox (*Urocyon cinereoargenteus*) rabies in west Texas, and preventing the reemergence of canine rabies from Mexico [11],[13].

Each ORV program is faced with multiple complexities [15], but none is as formidable as those associated with control of rabies in the raccoon. As an ecological generalist, raccoons often occur at extremely high population densities along the rural-urban interface [16] in common with the distribution of this rabies virus variant. Indeed, a major underlying impetus for the integration of ORV into conventional prevention and control beginning in the 1990s was the potential for accrued public health benefits of preventing continued spread of raccoon rabies within the eastern US [17] where much of the country's human population resides [18]. In turn, the increased chance for human-raccoon interactions and the difficulties of conducting control programs in the suburban mosaic along this interface poses unique and diverse challenges to rabies control.

This paper draws largely from specific examples of contemporary wildlife rabies control programs in North America to illustrate key ecological, biological, logistical, and environmental complexities and challenges, and the initiatives taken to achieve success.

## Methods

Literature searches included the use of Scopus via access through the USDA National Agricultural Library, and EBSCO Host Academic Search Premier via the University of New Hampshire's Dimond Library. Assistance in publication acquisition was provided by the USDA, Wildlife Services National Wildlife Research Center Library staff. Keyword searches included the following terms: *rabies*, *wildlife and rabies*, *ORV*, *TVR*, *rabies host shift*, and others.

### North American Collaboration Approaches

A broad scientific, regulatory, and management interface exists among the public health, agriculture, and wildlife management agencies responsible for specific rabies prevention and control activities in the US. Each state and federal agency has statutory authority and a public trust niche to achieve specific agency missions. As a consequence, planning, implementing, and coordinating effective rabies prevention and control necessitates inter-jurisdictional collaboration among diverse disciplines and authorities.

A national rabies management team approach has been applied in the US since 1999 to facilitate coordination of ORV programs across state and international boundaries in North America targeting specific rabies virus host reservoirs. The National Rabies Management Team is currently composed of nine smaller working groups focused on key topic areas such as surveillance, vaccine development, rabies control strategies, and research prioritization. The team meets annually to assess and discuss key issues and provides guidance for a spectrum of national wildlife rabies prevention and control goals [15]. The approach taken to assure science-based wildlife rabies control in the US is embodied in the “One Health Initiative” [19], a renewed global strategy recognizing that human and animal health, including wildlife, are inextricably linked. To effectively address infectious zoonotic diseases like rabies requires expanded interagency communication and collaboration. This national approach has been used as a springboard to develop a continental framework for rabies prevention and control in North America.

The signing of the North American Rabies Management Plan (NARMP) in October 2008 by representatives from Canada, Mexico, the US, and the Navajo Nation extends collaboration across national boundaries and multiple disciplines in four focus areas: communications, surveillance, control, and research. A fundamental tenet of the NARMP is that rabies prevention and control programs can be enhanced through an international collaborative framework. The formalization of this plan has spawned several proposed collaborative initiatives for 2009. Examples include a comparison of ORV performance between New Brunswick, Canada, and Maine, US, and closer coordination between border states and provinces involved in raccoon rabies control. Other examples include replication of the first ORV campaigns targeting dogs in Mexico, improved enhanced rabies surveillance along the Mexico-US border, and captive studies with the GnRH immunocontraceptive GonaCon in Mexico [20].

### Value of Enhanced Surveillance to ORV

Rabies surveillance is traditionally based on human or domestic animal exposure events brought to the attention of public health officials [12]. This system is effective in providing surveillance data to support critical medical decisions to protect public health. However, exposure-based surveillance tends to be biased by human population densities, political enumeration units, and other factors. This approach may not include a sufficient sampling scope or intensity to accurately delineate the leading edge of specific rabies virus variant distributions in real time or with the relatively high degree of confidence required for the timely and effective allocation of resources to ORV.

Enhanced rabies surveillance to support ORV in the US focuses on the following types of samples: strange acting (extremely aggressive or docile) animals where no human or domestic animal exposure has been reported, road kills, animals found dead in addition to road kills, animals with injuries or lesions indicative of highly aggressive behavior, and euthanized animals from focal trapping at sites where rabid animals were recently confirmed. Not uncommonly, raccoons captured by nuisance wildlife control operators are included as enhanced rabies samples [21], particularly when they can help delineate rabies distribution where control is being planned in suburban settings.

**Figure 1:**
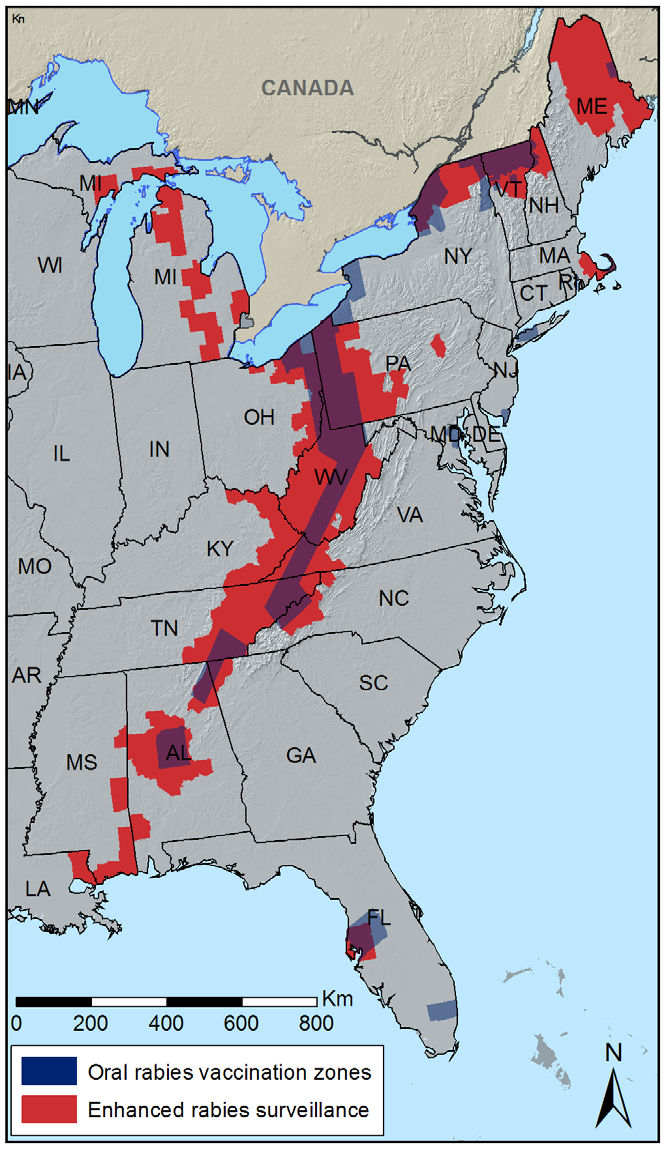
Raccoon oral rabies vaccination and Wildlife Services' enhanced surveillance counties in the United States of America, 2007.

The enhanced surveillance zone for raccoons typically extends from the areas where raccoon rabies is enzootic, through the ORV zone, to approximately 80 km into areas suspected to be free of raccoon rabies (Figure 1). The spatio-temporal distribution of animals tested for rabies is subsequently mapped using RabID (a geographic information system [GIS] database and internet mapping application) [22] such that near real-time decisions can be made to increase surveillance in areas, adjust ORV zones, or implement other rabies management actions such as trap-vaccinate-release (TVR).

Substantial increases in suspect rabid animal samples under the enhanced rabies surveillance protocol often create an undue burden on state laboratories for timely diagnosis. Application of a direct rapid immunohistochemistry test (dRIT) developed at the Centers for Disease Control and Prevention (CDC) in the US [23],[24] has permitted trained biologists in several states to mitigate much of this burden, while achieving the goal of expanding the geographic scope and intensity of rabies surveillance. In 2007, Wildlife Services (WS) tested 7,737 animals using the dRIT to enhance surveillance of raccoon rabies; 167 tested positive. In some states (e.g., New York), enhanced surveillance samples continue to be tested in a timely fashion by the state health rabies laboratory using the direct fluorescent antibody (dFA) test and virus typing by a US national protocol [25].

**Figure 2:**
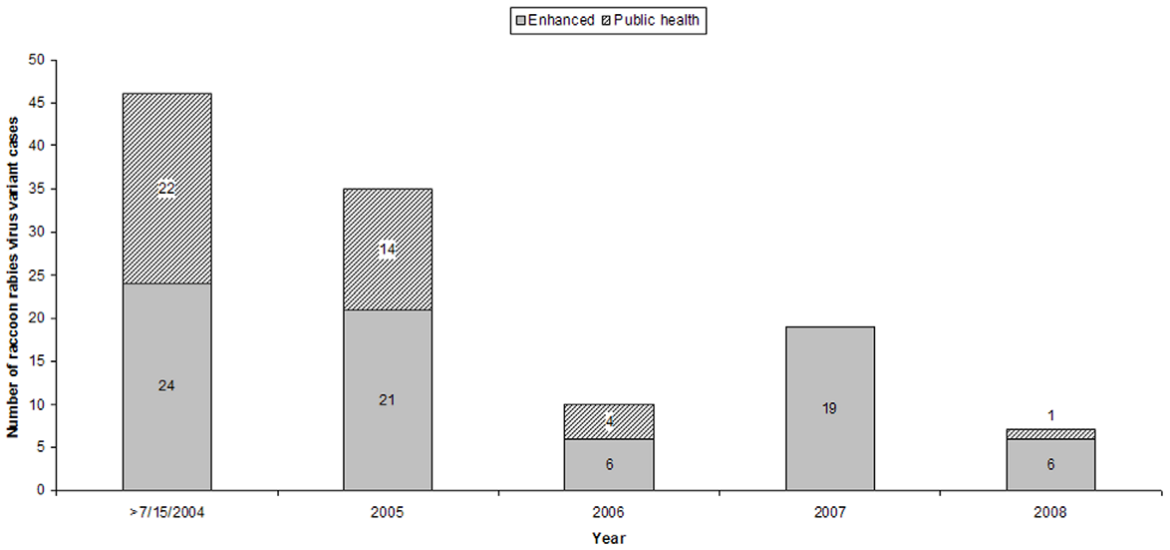
Raccoon variant rabies cases in Ohio contingency action zone. Since 16 July 2004 when raccoon rabies virus variant was first detected west of the existing oral rabies vaccination zone, 117 animals have been confirmed positive with raccoon variant within the contingency action zone. In 2007, no cases were detected via the public health surveillance system, illustrating the need for enhanced rabies surveillance.

**Figure 3:**
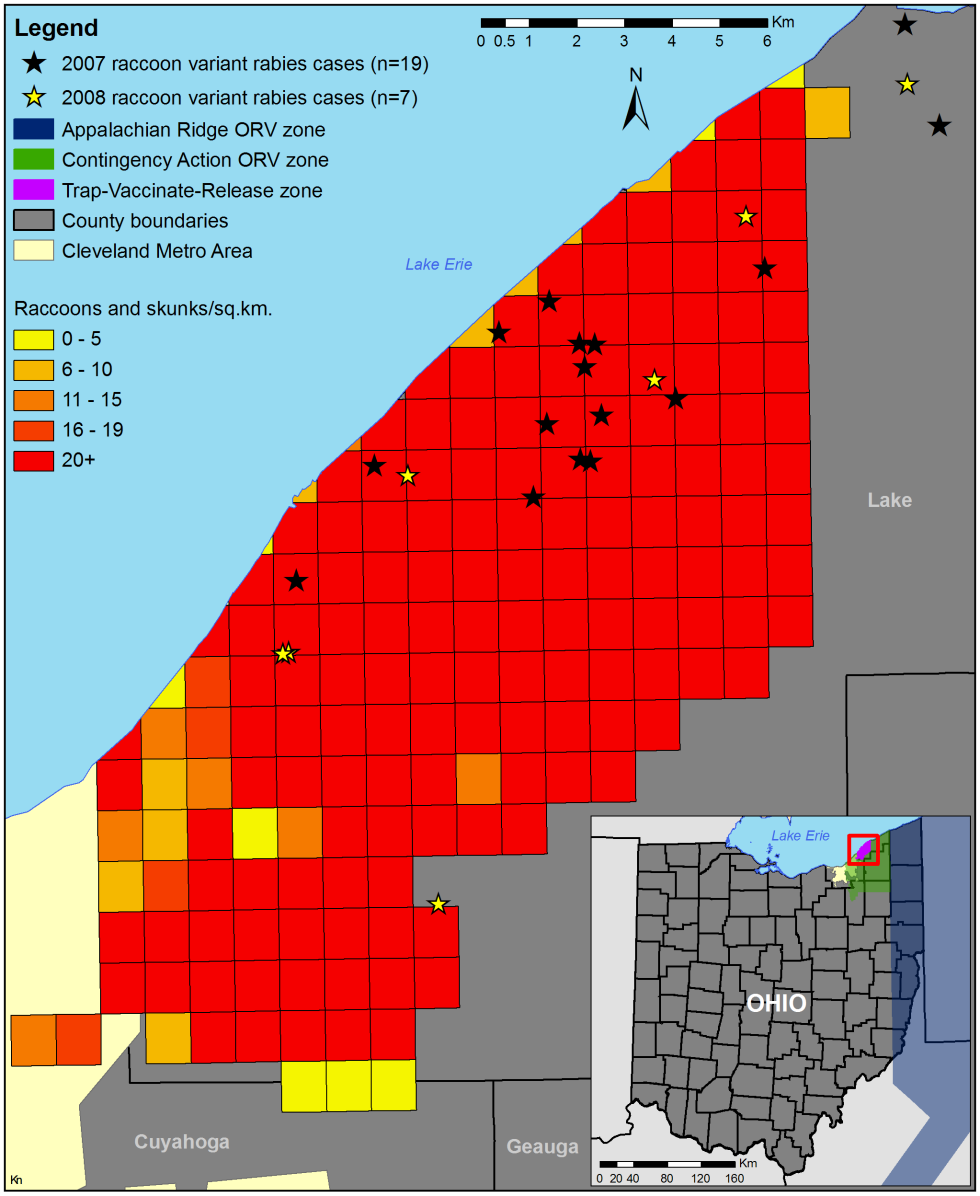
Contingency actions in Ohio, 2008. A large-scale trap-vaccinate-release (TVR) operation was conducted near Cleveland, Ohio, in 2008 and resulted in the hand vaccination of 4,196 raccoons and the brainstem testing of 138 raccoons and 77 skunks. The TVR zone consisted of 185 cells (1 km^2^ in size) and was delineated from raccoon variant rabies cases confirmed in the Contingency Action ORV zone in 2007 (*n* = 19). In addition to TVR, oral rabies vaccine baits were distributed over the area as part of contingency actions in 2008.

The value of enhancing rabies surveillance beyond levels typically conducted for the protection of public health was illustrated in northeast Ohio during 2007. This area is characterized by heavy commercial business and residential developments typical of suburbia (Cleveland metropolitan area). Since 2004, when rabies was first detected in this area (10 km west of the existing Appalachian ORV zone at that time), surveillance has been enhanced resulting in WS submitting 5,554 animals (through 2008) for rabies testing. In each year except 2007, rabies positive cases were detected through the public health surveillance system as well. From 2004–2006, there had been declining rabies cases, suggesting a trend toward rabies-free status in this contingency action area. In 2007, all rabies positive cases in this area were detected as a function of enhanced surveillance (Figure 2). This was a somewhat surprising result given the opportunity for exposure and presence of rabies in this highly developed suburban setting, underscoring the value of enhanced surveillance as a complement to traditional public health surveillance. The distribution of these positive cases (Figure 3) was critical to obtaining emergency funding and formulating a focused TVR campaign to bolster population immunity in an attempt to restore the area to raccoon rabies-free status.

### Raccoon Rabies ORV

Raccoon rabies was first described in Florida in the 1940s [26], and until the mid-1970s remained confined to the far southeastern US, extending north into South Carolina and west into Alabama [27]. Purposeful human-assisted translocations of raccoons to portions of western Virginia and southern West Virginia documented in the 1970s resulted in the creation of a new “Mid-Atlantic” raccoon rabies epizootic focus [28],[29]. Lack of access to ORV at the initiation of the outbreak or other practical methods that may have been applied on a landscape scale prevented intervention around the original focus. Unchecked, this unique virus variant spread rapidly in the ubiquitous and abundant raccoon populations of eastern North America. Thus, it has come to occupy its current expanded range from southwestern Alabama to the Maine–New Brunswick, Canada border and west to northeast Ohio, with emergence into southern Quebec (north of the Vermont border) in 2006 [12].

**Figure 4:**
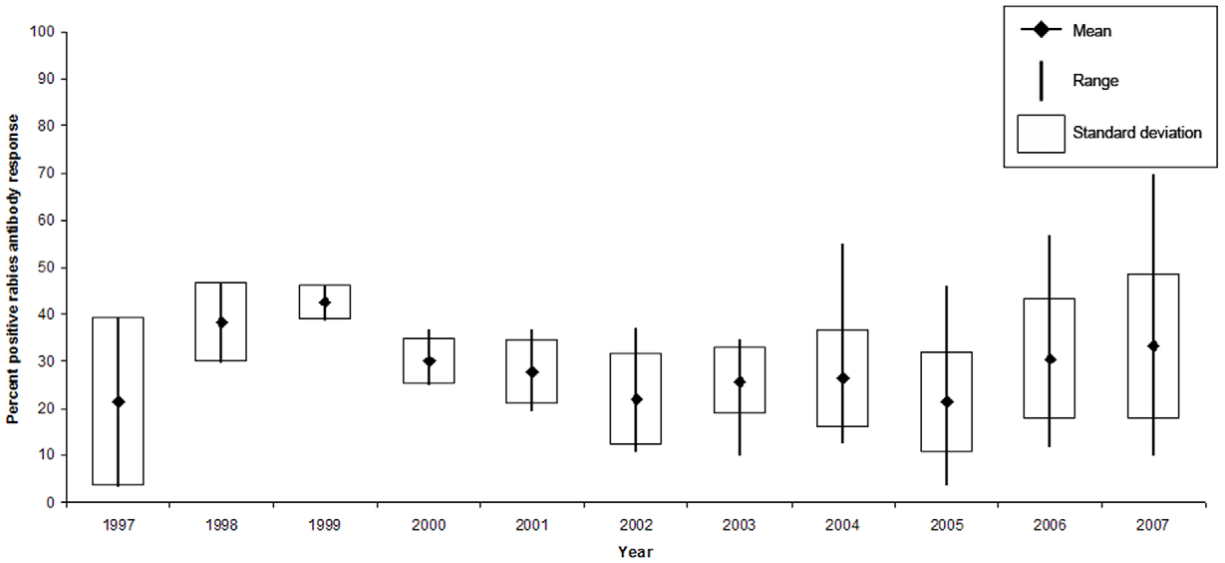
Percent positive rabies antibody response (≥0.05 IU).

During the 1990s, coordinated operational raccoon rabies control programs in the US expanded from small scale projects in five states (Florida, Massachusetts, Maryland, New Jersey, New York) to include portions of 16 states by 2005 [30]. A series of ORV zones have been strategically created to prevent raccoon rabies from spreading to unaffected areas. Mean post-ORV antibody activity (≥0.05 IU) for participating states as an index to population immunity for raccoons has averaged around 30% in these zones (Figure 4), with 2007, the most recent year with complete information, at 33.2%±15.5% (standard deviation). These observed antibody levels are low in comparison to the long-term annual average reported for gray foxes (61%) and coyotes (63%) [11] in states using the same oral rabies vaccine and similar baiting strategies. Nevertheless, raccoon rabies has not spread appreciably beyond ORV zones where abundant susceptible raccoon populations exist [31],[32] based on the expected rate of movement as observed in the eastern US [33], or as modeled for Ohio by Russell et al. [34], in the absence of ORV intervention.

Obviously, lower indices to population immunity connote a greater risk of ORV zone compromise and a reduced ability to sustain effective programs, highlighting the need for improved or new baits and vaccines and strategy refinements. Frequent spillover of raccoon rabies virus variant into the striped skunk [12] represents an additional confounding factor for long-term success [35], as essentially no antibody response has been observed in serum samples collected from skunks within ORV zones targeting raccoons [21]. Both factors have contributed to the need to apply contingency actions to bolster ORV campaigns to achieve rabies management goals. For example, use of population reduction, TVR (effective in skunks) and ORV in point infection control (PIC) in Ontario [36],[37], and a modified PIC in New Brunswick (M Allan, personal communication) have been credited with restoring both provinces to raccoon “rabies-free” status. In 2006 and 2007, PIC was conducted in southern Quebec to try to prevent raccoon rabies that emerged north of the ORV zone in Vermont from spreading north to Montreal and other human-occupied areas (P Canac-Marquis, personal communication).

In the US, a contingency action has continued in northeast Ohio since 2004, when raccoon rabies emerged 10 km west of the established ORV zone [15]. The strategy included ORV baiting by hand and by air twice/year (often at bait densities of up to 150 baits/km^2^), intensive enhanced surveillance, and TVR. In 2008, 4,196 raccoons were parenterally vaccinated in treatment cells (Figure 3), based on a priori raccoon population density indices, to achieve a targeted 65% vaccination rate. Additionally, 138 raccoons and 77 skunks were sampled and tested for rabies. Post-treatment serum analysis is in progress. Those results, in tandem with continued enhanced surveillance information, will be used to determine raccoon rabies status for the area and whether resources applied to this effort can be reallocated to other ORV priorities.

Contingency actions are an integral component of rabies management strategies in meso-carnivores to address local emergencies that may arise during normal ORV operations. However, such actions are labor intensive and wrought with logistical and environmental challenges that mandate careful coordination with the attendant high cost per unit area treated in comparison to regularly planned ORV campaigns. Comprehensive economic analysis of the benefits and costs associated with the 2008 contingency action in Ohio are in progress and will be compared to earlier reported estimates from Ontario that suggest the costs are approximately 2.5 times greater than ORV for a similar area [36]. Costs alone preclude their sustained application on a landscape scale or at a high frequency, but contingency actions remain important to address specific high-risk rabies foci to restore or maintain the integrity of larger ORV campaigns.

Key among the many challenges implicit in rabies control is the pervasiveness of the translocation of raccoons and other meso-carnivores [38], intentional or accidental, which represents a common extrinsic threat for both local and long-range movement of rabies and control programs [27]. Although translocation has been instrumental in specific endangered species recovery efforts and for other conservation purposes, large numbers of raccoons, skunks, and other species are purposefully moved about the landscape as a part of rehabilitation and nuisance wildlife control efforts [38]. Raccoons and other animals that commonly feed on household garbage may be unintentionally relocated considerable distances in transport vehicles associated with interstate movement of more than 42 million tons of municipal solid waste each year [39]. The wildlife profession and others now question translocation as a management tool in light of the need to contain or eliminate high-profile, economically important wildlife diseases, including rabies. Future plans will likely require a strategy that integrates at a minimum outreach education and revised laws, accompanied by enforcement, to make measurable inroads toward curbing this widespread problem.

### Vaccine-Bait-Biomarkers Needs

Adaptive methods for enhanced effectiveness in rabies control require attention to a broad range of research needs. These include ecology of reservoir species, an understanding of target and non-target species foraging behaviors, community dynamics of the meso-carnivore complex, bait uptake relative to a suite of species-specific spatio-temporal variables, and model development to support ORV decision making. Chief among these is the need for improved or new baits and vaccines that lead to enhanced field performance in raccoons and other species to reduce dependency on contingency actions and allow a shift in focus toward elimination strategies. This need is driven in part by rabies virus spillover and establishment among several sympatric meso-carnivores in North America [6],[13],[31]. Notably, no single vaccine has proven efficacious under field conditions for all relevant species. The community dynamics that lead to virus spillover events and subsequent host shifts are not well understood nor have the co-adaptive ecological relationships of the virus-host been extensively studied [13],[40],[41]. Nevertheless, spillover events and disease emergence present a challenge to the achievement of rabies control objectives in the absence of effective oral vaccines for use in the spillover host.

Notable examples of this dilemma have been documented in Alaska and Canada, where arctic fox rabies virus variant has spilled over into other animals, such as dogs and red foxes [42]–[44]. Infection of the latter species is especially problematic, because of the broad geographic distribution of red foxes extending from the arctic through the temperate zone, leading to subsequent spillover and maintenance in the striped skunk [6]. Previously discussed raccoon rabies virus variant spillover into striped skunks in the eastern US, as well as a host shift of big brown bat (*Eptesicus fuscus*) rabies virus to skunks near Flagstaff, Arizona, are other major examples [45]. Clearly, enhancement of existing oral rabies vaccine-bait combinations or the development of new products with proven field effectiveness in the striped skunk as well as the diverse complex of meso-carnivore rabies reservoir species in North America is needed.

In Ontario, field trials were conducted recently with a human adenovirus recombinant vaccine (ONRAB) developed with the intent that its strategic use will facilitate the elimination of the fox rabies foci maintained by skunks in southern Ontario [9]. Field trials with ONRAB have also been extended to raccoon rabies control in Ontario and Quebec. Several other new vaccine constructs have been developed that may hold promise for use in wildlife and dogs [46],[47]. For example, a canine adenovirus (CAV_2_) recombinant rabies vaccine may hold promise based upon preliminary results obtained in captivity upon a variety of species [48]–[52].

Tetracycline has had a long history as a biomarker in wildlife management, including extensive use in evaluating bait uptake in ORV campaigns [4],[53],[54], and its utility is well documented [55]–[57]. Disadvantages, particularly for ORV in raccoons, is that tooth extraction is intrusive, time consuming, and requires anesthesia, as live trapped raccoons are released at their site of capture once biological information and samples have been collected. In addition, first and second premolars have much lower tetracycline deposition rates than canine teeth, an unacceptable candidate sample for extraction from live animals. Because of this, tetracycline marking rates in raccoon premolar teeth from bait consumption are variable and do not always exceed sero-conversion rates as expected, compromising its usefulness as an index to bait uptake. Contemporary issues associated with antibiotic resistance and the uncertainty of tetracycline use in the future obliges us to seek out effective, environmentally compatible alternative biomarkers. Rhodamine B is one candidate under study as a potential post-bait ingestion marker, which shows promise in providing a short-term marker in growing whiskers as well as long-term in teeth. Further study is required to assess its field-level utility [58] as well as the feasibility of producing baits or vaccine that incorporate Rhodamine B.

### Rabies Control Intervention Revisited

In 2001, apparent spillover of big brown bat rabies virus variant resulted in 19 documented cases in skunks near Flagstaff, indicative of transmission among skunks [45]. Subsequent intervention with TVR has resulted in periods of quiescence followed by reemergence in 2004 and 2005 [21], and again in 2008 [59]. From 31 October 2008 – 1 May 2009, 17 cases of the same big brown bat variant were confirmed in gray fox, a species capable of longer range movement that could facilitate establishment of this variant in foxes and skunks over a broader geographic area [60],[61]. While these events support the hypothesis of a viral host shift from bats to carnivores, they also raise questions about the potential relationship between human perturbations that contributed to a locally abundant skunk population near Flagstaff and the development of this situation. The more immediate challenge is to determine if a practical and effective rabies management strategy can be formulated and funded to contain this apparently geographically limited focus. Elements of an effective strategy under consideration include ORV of sufficient scale to contain disease spread in gray foxes, periodic TVR in skunks until an effective oral bait-vaccine combination is available, and more comprehensive enhanced surveillance. The latter is to better understand the temporal emergence and spatial spread of bat rabies to susceptible carnivore populations. Failure to act while this focus appears to be largely restricted to the Flagstaff vicinity could result in the establishment of new terrestrial rabies variants over a broader landscape that would present an even greater challenge if control programs are contemplated in the future.

### Other Wildlife Rabies Control Considerations

The cost effectiveness of the shifting paradigm toward controlling rabies in meso-carnivore reservoirs should and will receive continual scrutiny. To date, studies of the benefits and costs have concluded that under specific assumptions and scenarios, ORV is a viable rabies management alternative [17], [62]–[65]. Ultimately, sustainability of programs in a highly competitive environment for government funding will hinge on achieving measurable successes on finite timelines. The bait-vaccine unit alone accounted for 88% of the ORV distribution materials and services' costs in the US, which included fuel and air contracting for operations targeting raccoons at bait densities of 75/km^2^ during fiscal year 2008. Consequently, accelerated emphasis toward the development and production of low cost, highly effective baits and oral vaccines that can clear the regulatory process for broad scale use is a critical need. Concomitantly, improvements to the currently licensed bait-vaccine appear warranted if effectiveness can be enhanced while cost/units are conserved. In turn, costs may be conserved by refining baiting strategies based on validated models that point to areas where reduced baiting density can be applied without sacrificing effectiveness [66]. Past economic analyses should be revisited to incorporate important new findings such as better estimates for benefits and costs of contingency actions and use of more powerful GIS analytical tools.

Concerns linger among some wildlife managers over potentially negative consequences from rabies control to species of conservation concern [67]. The unique rabies virus variant adapted to raccoons is a relatively new mortality factor, particularly in the northeastern US and portions of southern Canada, having reached southern New York in 1990, with an incursion into southern Ontario in 1999, New Brunswick in 2000, and Quebec in 2006. The ecological dynamics of rabies in relation to other raccoon disease mortality factors, such as canine distemper, have not been extensively studied to determine if the effects on populations are compensatory or additive. Clearly, additional study on this topic is warranted from an ecological and management perspective. Regardless, if raccoon predation is having an impact on bird nesting species or other species of concern [68],[69], dependence on a solution that implies reliance on the establishment of an unpredictable fatal virus, with tangibly serious public and domestic animal health implications, represents a dubious alternative to active management.

## Summary

Establishment of a rabies management team composed of a coalition of diverse expertise from the public health-agriculture-wildlife management interface has been critical to facilitate coordination among rabies control programs targeting meso-carnivores in the US. The NARMP has established a continental framework that extends collaboration and coordination, capacity for rabies communications, surveillance, control, and research among Canada, Mexico, and the US. Enhanced surveillance as a complement to public health surveillance has improved decision-making capability regarding allocation of rabies control resources, including contingency actions to address emergencies, as illustrated in Ohio. Raccoon rabies has not spread appreciably since ORV intervention has expanded in the eastern US, yet rabies virus neutralizing antibody levels in raccoon populations as an index to immune buffers in existing ORV zones point to the need for improved or new baits, oral vaccines, and strategy refinements. Achieving advances that lead to improved field performance should allow for a more aggressive movement of ORV zones into raccoon enzootic areas. Measureable successes beyond containment would be expected to enhance program sustainability toward the goal of broader scale elimination of raccoon rabies and ultimately other meso-carnivore rabies virus variants. Economic analyses will remain integral to ORV planning and as a means to characterize successes in costs and benefits. Conservation concerns related to control programs cannot be ignored and require additional study to better understand the role of rabies and other diseases on the population dynamics of meso-carnivores, such as the raccoon.

**Key Learning Points**

1. ORV represents a socially acceptable methodology that has helped eliminate canine rabies from the US, and restricted the distribution of raccoon, arctic fox, and gray fox variants of rabies in North America.
2. An international rabies management team composed of experts from the public health-agriculture-wildlife management interface has been vital to the establishment of viable rabies control programs in North America.
3. ORV in the US remains focused on the raccoon variant of rabies, while work continues to contain and eliminate the gray fox rabies variant in west Texas, and prevent canine rabies from re-emerging into the US from Mexico.
4. Advances in enhanced rabies surveillance that relies largely on a direct rapid immunohistochemistry test have led to improved real-time management decisions for meso-carnivore rabies reservoir species in the US.
5. ORV-related dog research in the southwestern US has potentially broad application in developing countries, where most of the 55,000 human rabies cases per year occur as the result of dog bites.

**Key Papers in the Field**

- Lembo T, Niezgoda M, Velasco-Villa A, Cleaveland S, Ernest E, et al. (2006) Evaluation of a direct, rapid immuno-histochemical test for rabies diagnosis. Emerg Infect Dis 12: 310-313.
- MacInnes CD, Smith SM, Tinline RR, Ayers NR, Bachmann P, et al. (2001) Elimination of rabies from red foxes in eastern Ontario. J Wildlife Dis 37: 119-132.
- Rupprecht CE, Hanlon CA, Blanton J, Manangan J, Morrill P, et al. (2005) Oral vaccination of dogs with recombinant rabies virus vaccines. Virus Res 111: 101-105.
- Sidwa TJ, Wilson PJ, Moore GM, Oertli EO, Hicks BN, et al. (2005) Evaluation of oral rabies vaccination programs for control of rabies epizootics in coyotes and gray foxes. J Am Vet Med Assoc 227: 785-792.
- Velasco-Villa A, Reeder SA, Orciari LA, Yager PA, Franka R, et al. (2008) Enzootic rabies elimination from dogs and reemergence in wild terrestrial carnivores, United States. Emerg Infect Dis 14: 1849-1854.
